# A retrospective multicenter case series of real-world tralokinumab use in dupilumab-experienced patients

**DOI:** 10.1016/j.jdcr.2024.01.021

**Published:** 2024-02-01

**Authors:** Edward I. Herman, Jessica Burgy, Mona Shahriari

**Affiliations:** a South Shore Dermatology Physicians, North Easton, Massachusetts; b Columbia Dermatology, Columbia, South Carolina; c Yale University School of Medicine, New Haven, Connecticut; d Central Connecticut Dermatology, Cromwell, Connecticut

**Keywords:** atopic dermatitis, biologic therapy, body surface area, dupilumab, interleukin 4, interleukin 13, investigator’s global assessment, moderate-to-severe, multicenter case series, patient-reported outcomes, real-world evidence, tralokinumab

## Introduction

Atopic dermatitis (AD) is a chronic relapsing inflammatory skin disease that has a heterogeneous clinical presentation and a multidimensional burden of disease beyond the skin that negatively impacts patients’ quality of life.^1^ In fact, time spent managing AD is associated with increased disease burden, decreased quality of life, and negative mood changes.^2^^,^^3^ There remains a significant need for treatment options that offer patients long-term disease control with a favorable safety profile. Dupilumab, a monoclonal antibody that targets interleukin (IL)-4Rα, blocking signaling of IL-13 and IL-4, is approved for the treatment of children and adults with moderate-to-severe AD in multiple countries.^4^, ^5^, ^6^ Tralokinumab, the first fully human monoclonal antibody that specifically neutralizes IL-13, blocking its interaction with its receptor, is approved in multiple countries, including in the European Union, United Kingdom, Canada, and the United States, for adults with moderate-to-severe AD.^7^, ^8^, ^9^ Phase 3 trials showed tralokinumab provided significant improvements in the signs and symptoms of AD and was well-tolerated up to 52 weeks of treatment.^10^^,^^11^

Although dupilumab is highly effective for the treatment of moderate-to-severe AD, in some patients, it may not adequately control the signs and symptoms of AD or may result in adverse events (AEs) that necessitate its discontinuation. In these patients, a medication with a different mechanism of action may provide relief. Tralokinumab has a distinct mechanism of action by specifically targeting the IL-13 cytokine and is a reasonable option for biologic naïve patients as well as patients who have tried and failed dupilumab. However, real-world evidence evaluating the efficacy and safety of tralokinumab use in patients with moderate-to-severe AD who were previously treated with dupilumab is limited. Approximately 4% to 14% of patients with AD experience treatment failure with dupilumab as a result of either worsening AD or AEs, including conjunctivitis.^12^ Here, we report a retrospective multicenter case series of tralokinumab use in 9 dupilumab-experienced patients in the United States. These findings help to further characterize the efficacy and safety profile of tralokinumab.

## Methods

### Patients

Adult patients with moderate-to-severe AD from 3 dermatology practices in the United States, who were previously treated with dupilumab, and subsequently switched to tralokinumab, were included. The health care providers at these sites recorded clinical information from these patients as part of their routine clinical practice.

### Data collection

Baseline characteristics collected included history of previous treatments, comorbidities, morphologic and topographic AD phenotype, investigator’s global assessment (IGA) and body surface area (BSA) before and at the time of initiating tralokinumab treatment, disease duration, duration of dupilumab treatment, and reason for dupilumab discontinuation. Data collected related to tralokinumab treatment included duration of treatment, the dose administered, IGA, BSA, patient-reported outcomes (eg, itch, clearance of erythema, and treatment satisfaction), and AEs possibly related to tralokinumab.

## Results

### Baseline characteristics

Nine patients were included for the case series (see baseline characteristics in Table I). Eight patients (89%) reported experiencing plaques/classical AD, 6 patients (67%) reported previously using prednisone, and 5 patients (56%) reported having asthma as a comorbidity. Median (range) IGA at baseline and IGA at time of tralokinumab administration were 4 (3-4) and 3 (2-4), respectively. Median BSA at baseline and BSA at time of tralokinumab administration were 20% (4%-30%) and 10% (1%-30%), respectively. Disease duration ranged from 1 to 2 years to over 20 years. For 6 patients with available information, duration of dupilumab treatment was 2 to 8 months. Reasons for discontinuing dupilumab treatment included inadequately controlled AD on dupilumab (3 patients), AEs including conjunctivitis (3 patients), injection site reaction (1 patient), and joint pain (1 patient) that led to patient-initiated discontinuation of dupilumab, resulting in a flare of the AD. Median (range) time between stopping dupilumab and initiating tralokinumab was 1.5 (0.5-24) months; the interval varied due to several factors including the availability of tralokinumab samples in the clinic or waiting for insurance coverage to initiate tralokinumab.

**Table I:** Baseline characteristics and outcomes of 9 dupilumab-experienced patients receiving tralokinumab

Patient No.	Baseline characteristics at time of tralokinumab initiation									Outcomes on tralokinumab					
	Sex¶	Age (y)	Ethnicity	IGA	BSA (%)	Duration of AD (y)	Duration on dupi (mo)	Reason for dupi discontinuation	Time between stopping dupi and initiating tralo (mo)	Duration on tralo (mo)	Tralo dose	IGA	BSA (%)	Improvement in PROs	AEs
1	F	68	White	3	4	5	8	Inadequately controlled AD	1	3	Q2W	0	0	Clearance, happiness	None
2	F	65	White	3	28	>5	>5	Inadequately controlled AD	2	3.5	Q2W	2	8	Clearance, itch	None
3	F	65	White	4	20	7	Unknown	Inadequately controlled AD	24	2	Q2W	3	10	Clearance, itch	Mild scalp dermatitis
4	M	71	White	2	5	Childhood	6	Intractable conjunctivitis	1	6	Q2W	1	2	Itch, happiness	None
5	F	49	White	2	5	Childhood	4	Conjunctivitis	1	2	Q2W	0	0	Itch	Herpes labialis§
6	F	52	White	2	1	1-2	2	Conjunctivitis	0.5	3	Q2W	1	1	Happiness	None
7	F	50	White	3	10	1-2	4	Joint pain	1.5	8	Q4W	0	0	Happiness	None
8	F	85	White	3	25	7	Unknown	Injection site reaction	8	7†	Q2W	0	0	Itch and sleep	None
9	M	82	White	3	30	>20	Unknown	Possible arrhythmia∗	6	5‡	Q2W	0	0	Clearance, itch	None

*AD*, Atopic dermatitis; *AE*, adverse event; *BSA*, body surface area; *dupi*, dupilumab; *F*, female; *IGA*, investigator’s global assessment; *M*, male; *PRO*, patient-reported outcome; *Q2W*, every 2 weeks; *Q4W*, every 4 weeks; *tralo*, tralokinumab.

∗ Subjective palpitations shortly after initiation of dupilumab treatment.

† Patient was clear at 3-month follow-up.

‡ Patient was clear at 2-month follow-up.

§ Unclear if related.

¶ Defined as patient-reported biological sex.

### Outcomes on tralokinumab

**Fig 1:**
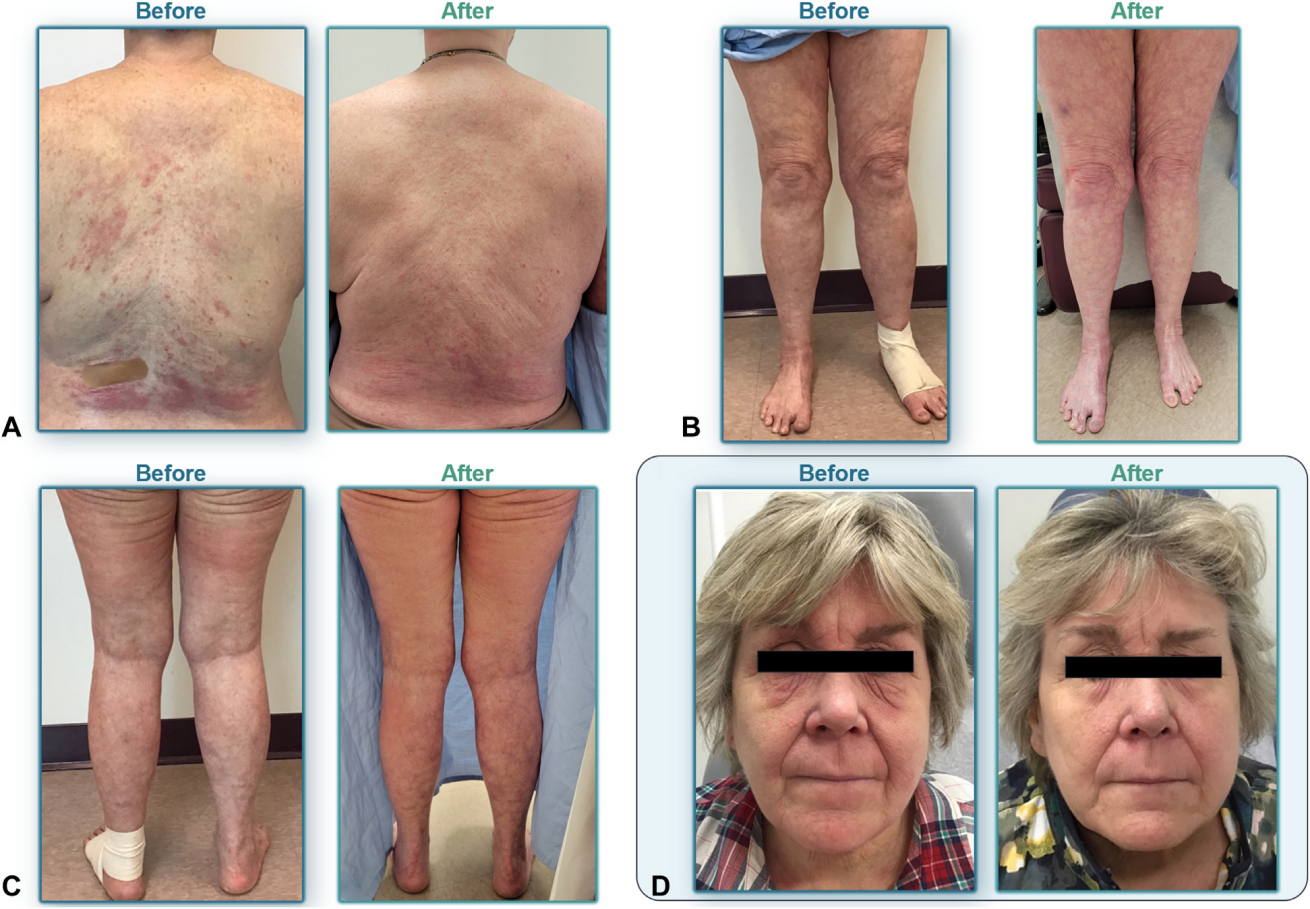
**A-C,** Photographs of dupilumab-experienced patients 2 and patient 3 (**D**) before and after initiating tralokinumab. (Patients provided consent for use of photographs. Previous treatments included methotrexate [stopped because of worsening fatigue after 4 to 5 years], dupilumab, upadacitinib [stopped because of increasing liver function tests and body mass index], prednisone, and phototherapy.)

All 9 dupilumab-experienced patients received the loading dose of 600 mg of tralokinumab and were then administered on-label tralokinumab (every 2 weeks for 8 patients, and every 4 weeks for 1 patient) and had been receiving tralokinumab for 2 to 8 months. As standard practice, all patients also had access to and were encouraged to use topical steroids as needed before and after initiating tralokinumab. At the time of data collection, median (range) IGA and BSA for the 9 patients were 0 (0-3) and 0% (0%-10%), respectively. Patients experienced improvements in measurable patient-reported outcomes and subjective satisfaction; 67% (6/9) of patients reported improvements in itch with NRS scores of 0 or 1, 44% (4/9) reported general clearance of AD signs and symptoms of AD, and 44% (4/9) reported their overall satisfaction of receiving tralokinumab. A summary of patient outcomes on tralokinumab can be seen in Table I. Improvements in patient-reported outcomes were reported and clearance of AD signs can be seen visually in patients 2 and 3 in Fig 1, *A*-*C* and Fig 1, *D*, respectively. AEs of conjunctivitis (3 patients) and joint pain (1 patient) completely resolved upon switching from dupilumab to tralokinumab. Residual signs and symptoms of AD following initiation of tralokinumab were managed with antihistamines (1 patient), prednisone (1 patient), or topical steroids. Patients exhibiting comorbid asthma did not show any apparent worsening of symptoms or flares while receiving tralokinumab. The patient who reported heart arrhythmia as a potential reason for discontinuing dupilumab treatment had a history of congestive heart failure but experienced no recurrence of cardiac symptoms or worsening of their congestive heart failure since starting tralokinumab. Only 2 patients reported AEs: (1) 1 patient with mild erythema and scaling of the scalp, which resolved after topical treatment and did not recur while the patient continued tralokinumab, and (2) 1 patient with herpes labialis, which was unclear if related to tralokinumab treatment.

## Conclusion

Although dupilumab provides clinical benefit for many patients, some patients struggle to achieve adequate control of the signs and symptoms of AD or experience AEs while while receiving dupilumab therapy. Dupilumab selectively binds to IL-4Rα, thereby blocking both IL-4 and IL-13 signaling. Tralokinumab, in contrast, binds to the IL-13 cytokine directly thereby allowing for normal IL-4 signaling. IL-13 is critical in the pathogenesis of AD and IL-13 blockade is sufficient to ameliorate AD, as evidenced by the efficacy of tralokinumab and lebrikizumab for moderate-to-severe AD.^11^^,^^13^ The impact of additionally blocking IL-4 in terms of efficacy and adverse reactions remains unclear. Although not mentioned in the original phase 3 clinical trial results, AEs such as dupilumab-associated inflammatory arthritis have emerged in the postmarketing period; these phenomena, as well as conjunctivitis, have been theorized to relate to the effect of IL-4 blockade “unmasking” or shifting the overall immune milieu toward the T helper 1 (Th1) or Th17 axis due to its critical role in Th2 differentiation.^12^^,^^14^^,^^15^ Although the immunologic basis for these effects is likely complex, it is reassuring that subsequent treatment with a selective IL-13 antagonist can avoid recurrence, at least in our case series.

The 9 patients presented in this case series provide real-world evidence of tralokinumab use in patients with moderate-to-severe AD who were previously treated with and failed dupilumab therapy. Tralokinumab and dupilumab have distinct mechanisms of action, and given the heterogeneity of AD, some patients may see improvement in their symptoms or resolution of AEs when switching from dupilumab to tralokinumab. In particular, we observed resolution of conjunctivitis and joint pain in patients who switched from dupilumab to tralokinumab. Our real-world findings are particularly useful for patients in which Janus kinase inhibition may not be ideal in the setting of dupilumab failure. Future studies, on a larger scale, can help us elucidate how differences in mechanism of action can lead to varying efficacy responses and safety signals and ultimately better outcomes for patients.

## Conflicts of interest

Dr Herman has been an investigator for Lilly USA, CorEvitas Atopic Dermatitis Registry, and CorEvitas Psoriasis Registry and has been a consultant (received honoraria) for Bristol Myers Squibb and Leo Pharma. Dr Burgy has been an investigator for Lilly USA, CorEvitas Atopic Dermatitis Registry, and CorEvitas Psoriasis Registry. Dr Shahriari has been a consultant (received honoraria) for AbbVie, Arcutis, Amgen, Bristol Myers Squibb, Dermavant, Janssen, Leo Pharma, Lilly USA, Novartis, Ortho Dermatologics, Sanofi-Genzyme, Regeneron, and UCB, has served as a speaker for AbbVie, Arcutis, Bristol Myers Squibb, Lilly USA, Janssen, Dermavant, Leo Pharma, Sanofi-Genzyme, and Regeneron; and has been and investigator for AbbVie, CorEvitas Psoriasis Registry, Dermira, Cara, Dermavant, Novartis, Union, Mindera.
